# Czech Consumers’ Preference for Organic Products in Online Grocery Stores during the COVID-19 Pandemic

**DOI:** 10.3390/ijerph192013316

**Published:** 2022-10-15

**Authors:** Martina Zámková, Stanislav Rojík, Martin Prokop, Simona Činčalová, Radek Stolín

**Affiliations:** 1 Department of Mathematics, College of Polytechnics Jihlava, Tolstého 16, 58601 Jihlava, Czech Republic; 2 Department of Management, Faculty of Economics and Management, Czech University of Life Sciences Prague, Kamycka 129, 16500 Prague, Czech Republic; 3 Department of Economics Studies, College of Polytechnics Jihlava, Tolstého 16, 58601 Jihlava, Czech Republic

**Keywords:** consumer behavior, organic food products, sustainable agriculture, food security and food safety, human environment, quality of life, COVID-19, health behaviors, sustainable development of rural and urban areas

## Abstract

A major advantage of online organic produce shopping is the fact that it saves energy and reduces emissions otherwise generated by customers during their time spent on the road and while shopping. Organic products in general positively impact sustainability, the environment, and the regions of their origin along with the social changes in these regions and further rural development. Moreover, these products positively impact the perceived health benefits and quality of food labeled as organic. The Czech Republic has currently seen a rise in organic food purchasing and supply trends. This study maps the factors possibly influencing consumers’ decision to go shopping for organic food online. Observed factors include the following demographic characteristics of consumers (respondents): gender, age, education, household income, number of children in the household and number of household members. A total of 757 respondents from the Czech Republic from September 2020 to December 2020 took part in the research. Logistic regression, used for data processing, identified the statistically significant effects of education, income and number of household members on online purchases. These conclusions were confirmed by a detailed contingency tables analysis, including the almost monotonous trend of the dependencies, with only minor deviations in a maximum of one category. The strongest influence of some categories on the emergence of partial dependencies was found by residue analysis. The research confirmed that the frequency of online grocery shopping increases significantly with increasing education and income of respondents and decreases with increasing the number of household members. Most respondents apparently shop for groceries online because of time savings, better product choice and more convenient and easier search.

## 1. Introduction

In recent years, the organic food retail business in the Czech Republic, as in many other European countries, has undergone a structural change [1,2]. This change is most distinct in the reduction of retail shops, the higher competition due to the emergence and the development of various new store formats [3,4]. Environmental and health problems have increased the interest of researchers and practitioners in investigating the factors that affect organic food and agriculture consumption [5,6,7], specifically e. g. problems of health and environmental impact of protein or minerals consumption etc.

The choice of a particular store type has been the subject of extensive research and has been studied from different perspectives. However, some consumers may still not have access to certain store concepts that they would prefer. For example, distance [8] or time constraints may prevent consumers from shopping in supermarkets, forcing them to use convenience stores instead, which are more suitable for smaller purchases [9]. When it comes to food, traditional brick-and-mortar stores still dominate as the main shopping channel. But nowadays they are being revitalized considerably, offering new services and also turning into pick-up points [10,11,12]. However, customers also want to support small (e.g., regional) businesses, unique products (e.g., organic products [13] or organic food) [14] and experience quality customer service [15], and they like to buy new brands [16] and visit new shopping sites [17]. Typical examples are markets where one can support local vendors or farmers, buy seasonal fruits or vegetables as well as fresh or dried herbs, spices, flowers, honey, and other locally produced products. According to [18], buying local organic food is an important economic and environmental reason for sustainable development of rural and urban areas and also health choice, e.g., protein sources.

New store formats are designed to meet the consumers’ needs, primarily by adapting the assortment, opening hours and shopping site [19,20]. New and different concepts give consumers more choice and flexibility [21]. Respondents of the research [22] prefer to buy organic food directly from producers, followed by supermarkets, specialty stores and pharmacies. The COVID-19 pandemic has impacted food supplies across the world, and public health restrictions have changed the way people shop for food, potentially exacerbating food insecurity [23,24]. Moreover, during the pandemic spread of COVID-19, according to [25], retirees, in spite of the digital divide, were also the ones that most increased online shopping.

As [26] says, changes in the retail sector and innovation over the last 30 years have led to the need to examine not only which store concepts consumers use for shopping, but also the means of their shopping. In expert studies, consumer segmentation has been based on different types of variables such as demographic [27], psychological [28], lifestyle factors such as environmental or health behaviour [29] or selection criteria such as price and food safety and quality or food or food composition like minerals, sugar, fat or protein or protein sources–plant based or animal protein etc. [30,31].

Gender plays a significant role in purchasing decisions. Until recently, food purchasing was mainly the domain of women, who were considered the main purchasing agents of the household according to [32]. However, modern times are causing changes in traditional gender roles in the household. There has been a significant increase in the number of men shopping as more women work out of their homes [33]. The authors of the study [34], who examined the American market, concluded that gender does not affect the purchase of organic food. One new type of male shopper emerged from research [35] that has not been found in the literature before. This shopper is presented as young, well-educated, early in his career and family life cycle, attracted by a strong brand offering and willingness to buy groceries for his family.

The study results [36] suggest that there is a relationship between the age of customers and frequency of online grocery shopping of the organic food. However, customers who shop for food online are still reluctant to buy perishable goods (meat, baked goods) and prefer healthy and environmentally friendly goods that are packaged directly by the manufacturer.

Another equally important demographic factor is age. As consumers get older, they demand high-quality products and services and are willing to put in the effort to meet their needs, according to [37]. Older people also tend to be more loyal. However, a negative experience that aging brings is the increased difficulty in coping with the changing store environment.

Income is another and one of the most important demographic factors that significantly influences consumer consumption, choice of store and also the amount of organic food purchased. According to [38], household income has a significant impact on its purchasing decisions. In general, it can be observed that people with higher incomes have attained a higher level of education [39], and people with a higher education usually need more information when making decisions [40]. According to [41], households with higher incomes are willing to buy organic food products more often. Conversely, people on lower incomes buy organic food less.

The conclusion reached by the authors of [42] suggests that relative proximity to home and lower prices are the main factors influencing grocery shopping in brick-and-mortar stores, while product variety, staff politeness, store atmosphere and household income level are the main factors that influence the preferences of consumers who shop for groceries in modern shopping malls. The authors also found that demographic factors such as the age, gender, and number of family members do not influence the choice of store (supermarket, mall, etc.). However, on the other hand, household size was found to be related to unplanned shopping, which is positively related to the size of the bill for groceries. If the household is big, consumption is high, which may indicate a relationship between food consumption and household loyalty. Current research confirms that family size has a positive effect on food purchases (e.g., [43]).

It is not only demographic characteristics such as age, gender and income, or factors related to residence that influence purchasing behaviour. The authors of [44] assessed the importance of four other factors influencing food choice (taste, price, health and convenience) and identified consumer segments according to their ratings. The results showed that age, gender, place of residence and who is responsible for grocery shopping were always positively related to involvement in the segments.

A more comprehensive study in this area [45] found that shoppers’ age, gender, education, occupation, monthly household income, family size and distance travelled to the store were significantly related to retail choice decisions. Choice decisions also vary by shoppers’ demographic attributes. The frequency of age and gender purchases of organic food was confirmed by a Chinese study [46]. In Bangladesh [47], age, education and household income also proved to be significant factors. On the other hand, gender and marital status did not affect the purchase of organic food. According to [48], the socio-demographic characteristics have nothing to do with the willingness to buy organic food.

Grocery shopping is an integral part of human life. We spend up to several days a year doing this activity. Saving time, energy and the environment are increasingly valuable to customers and their health. The economic crisis and the increasing penetration of digital technologies have also triggered significant changes in shopping habits. In recent years, there has been a growing interest in the topic of online shopping not only from consumers but also from researchers (e.g., [49,50,51]) and this expansion can also be expected in the future. The aim of our paper is to identify a profile of a typical online grocery shopper focusing on organic foods that are becoming increasingly popular [52,53].

The main motivation of the study is the online convenience of shopping, and the ability to save time and effort motivates convenience shoppers to shop online [54]. Shoppers save energy and fuel as well [55] as their health during the COVID-19 times [56]. The online shopping covers convenient needs, safety needs, variety needs and health needs like i. E. special customer´s preferences in protein -based diet, plant based protein or general protein sources and environmental impact label etc. [57].

## 2. Materials and Methods

A total of 757 responses from surveyed respondents in the Czech Republic were included in the research for further processing. This is a representative quota sample. The quotas used are shown in Table 1. Respondents were contacted between September 2020 and December 2020 via an online questionnaire. The attitudes of respondents and their grocery shopping behaviour were investigated, with an emphasis on online grocery purchases. The main research question–whether customers shop for groceries online–was chosen due to COVID restrictions and related mobility constraints of the population. The research data were analysed in relation to basic socio-demographic characteristics on a selected sample of respondents in the Czech Republic. In order to find out the consumer profile of a typical customer, identification variables were chosen—gender, age, education, household income, number of children in the household and number of household members. These identifiers were specifically chosen as they are considered the most important according to the literature review, e.g., [38,42,45,46,48]. More women were selected for the research because historically, women have been more likely to fulfil the role of food shopping [58]. Moreover, in the area of incomes, a larger representation of customers with wages between 30,001–40,000 CZK was selected because in 2020 the median of wages in the Czech Republic is in this interval [59].

The mathematical model describes how whether a consumer buys organic food online depends on selected socio-demographic indicators. The model contains categorical variables, and therefore methods for processing categorical data were used to determine the consumer profile of organic food buyers, i.e., logistic regression, analysis of contingency tables and correspondence analysis. These methods are used in the analysis of customer preferences and consumer behavior research [60,61,62,63]. The results were presented by means of correspondence maps. The logistic regression maps the dependence of the studied variable (Do you buy groceries online?) on several selected socio-demographic variables simultaneously. The contingency tables are then used to track in more detail the partial dependencies of the research question on each of the identifying variables. The selected analyses can be used from a marketing perspective to define and refine the target group and to create a profile of the target customer group [64,65].

The parameters of the logistic regression model are estimated using the maximum likelihood method. Wald statistics are used to test the significance of the regression coefficients. The quality of the model is evaluated e.g., by the chi-square goodness-of-fit test, with the value of the chi-square statistic being multiplied by −2 [66,67,68].

For the binary explanatory variable (Do you buy groceries online?), factors that have a significant effect on whether the respondent buys organic food online (coded as 1) or does not use this option at all (coded as 0) were analysed. The explanatory variables gender, age, education, income, number of members and children in the family, and size of the municipality were considered as categorical variables (the variables age and income were interval-defined), and the values of the variables were coded with an increasing ordinal scale of 0, 1, 2, … corresponding to the increasing value of the variable. The nominal gender variable was then scaled by values of 1 (female) and 0 (male). By successive testing of models containing explanatory variables, the gender, age, number of children in the household and size of the municipality variables were dropped as their effect proved to be insignificant.

Contingency table analysis, including the Pearson’s chi-square test, was used to analyse in detail the dependence of the studied variable (Do you buy groceries online?) on each identifying variable separately; for more details, see [69,70].

According to [64], correspondence analysis is applied, e.g., in the field of marketing research in the evaluation of consumer behaviour. Correspondence analysis shows the correspondences of categories of individual variables and provides a common picture of row and column categories in the same dimensions. Unlike most other multivariate methods, correspondence analysis allows for the treatment of categorical non-metric data and non-linear relationships. It is analogous to factor analysis, but instead of factors, the influence of individual categories, their mutual similarity or association with categories of other variables is observed [71,72].

Software STATISTICA and UNISTAT were used for the processing of primary data. The usual value of 0.05 was chosen for the significance level. 

## 3. Results

The results of the research show that 80.58% of the surveyed sample of customers buy organic food online. For the binary variable (Do you buy groceries online?), the factors that have a significant effect on whether or not the respondent buys organic food on the internet were analysed. Using logistic regression, statistically insignificant variables were removed from the identification questions—gender, age, education, income, number of members and children in the household, and size of municipality. There was no dependence on gender, age, income and number of children in the household. The parameter estimates of the resulting regression model, including the values of the Wald statistic, the significance of the individual coefficients and the 95% confidence intervals, are presented in Table 2, which shows the significant dependence of online grocery shopping on the respondent’s highest level of education, the respondents’ net monthly household income and the number of household members of respondents’ family. The research results show that on average, the frequency of online grocery shopping according to the sign of the regression coefficients shows an increasing trend for those with higher education and higher income, while the frequency of online grocery shopping decreases as the number of household members increases. The model is statistically significant, *p*-value < 0.0001.

In addition to the overall model, due to the need for a more detailed description of the partial dependencies, a contingency table was created for each individual dependency, and the existence of the dependency was verified by Pearson’s chi-square test. The intensity of the dependence was measured by Pearson’s contingency coefficient. The effect of each category on the explanatory variable was examined by residual analysis.

The increasing trend in the frequency of online groceries shopping with higher education is evident in Table 3. The intensity of the relationship is lower given the 2 degrees of freedom; the Pearson’s coefficient is 0.11. The coefficient is statistically significant. The frequency of online grocery shopping increases with the higher education of the respondents. The respondents who reported their highest education as without GCE (77.42%) purchase groceries online the least frequently, the respondents with secondary education with GCE (80.92%) purchase groceries online a little more frequently, and the respondents with the highest education (88.30%) shop for groceries online the most.

The post hoc residual analysis can be seen in Table 4. At the 5% significance level and in the case of six cells in the contingency table, the residue cut-off value is 2.638. Residuals greater than 2.638 in absolute value indicate the categories that contribute most to the dependence. Table 4 shows that the categories of respondents with a university education contribute most strongly to the fact that respondents buy organic food online, while the category of respondents with the lowest education is also on the borderline.

The majority increasing trend in the frequency of online grocery shopping with higher monthly family income can be seen in Table 5. Thus, it is clear that the higher the net monthly income of the respondents, the more frequently they buy organic food online. The intensity of the relationship is moderate given the 4 degrees of freedom; the Pearson’s coefficient is 0.13. The coefficient is statistically significant. The frequency of online grocery shopping increases with the higher income of respondents; the least frequent purchases are made by respondents with income up to 20,000 CZK (71.88%), and the most frequent purchases are made by respondents with income of 50,001 CZK and more (86.24%).

The results of the post hoc residual analysis can be seen in Table 6. Residuals greater than 2.807 in absolute value indicate the categories that contribute most to the fact that respondents shop for organic food online. At the 5% significance level and for 10 cells in the contingency table, the cut-off value for residuals is 2.807. Table 6 shows that the categories of respondents with the highest and lowest income are closest to the significance level and thus have the greatest influence on the emergence of dependence.

Consumers with more than four household members shop for groceries online least often (68.48%). Single-person households are the most likely to buy organic food online (89.47%). Thus, the results show a majority decreasing trend in the frequency of online organic food purchases as the number of household members increases; see Table 7. The intensity of the relationship is moderate given the 4 degrees of freedom; the Pearson’s coefficient is 0.13. The coefficient is statistically significant.

A post hoc residual analysis can be seen in Table 8. At the 5% significance level and for the 10 cells in the contingency table, the cut-off value of the residuals is 2.807. Residuals greater than 2.807 in absolute value indicate the categories that contribute most to the fact that respondents shop for groceries online. Thus, the categories contributing most strongly to dependence are respondents from families of five or more, who shop for groceries on the Internet significantly less frequently.

The correlation coefficient was calculated for the variables number of household members and number of children in the family due to the assumption of possible collinearity (too strong linear dependence) and its value is 0.68. Its value is therefore lower than the usual cut-off value of 0.8 indicating collinearity. Thus, despite the stronger dependence, both variables were included in the original regression model.

The variables such as gender, age, number of children in the family and size of the municipality where the respondent lives, which were removed from the regression model due to insignificance, were also analysed for their effect on the fact that respondents buy organic food online. Contingency table analysis was used to process these variables. This analysis shows that the proportion of respondents buying groceries online is not significantly related to gender when using a chi-square test as well as when using logistic regression. About 80% of both men and women occasionally buy groceries online. The dependence of the fact that a respondent shops for groceries online on the age of the respondent is also not statistically significant, but some differences do exist. Nearly 90% of respondents in the 26–35 and 36–45 age categories shop for groceries online, while only less than 80% of younger or older respondents do so. The proportion of respondents shopping for groceries online does not initially decrease much as the number of children in the household increases (around 80%) until it drops to 70% for households with three children and 60% for families with four or more children. Using a chi-square test, this relationship is statistically significant. The proportion of respondents buying organic food online does not show a clear trend dependency on the size of the municipality in which the respondent lives.

**Figure 1:**
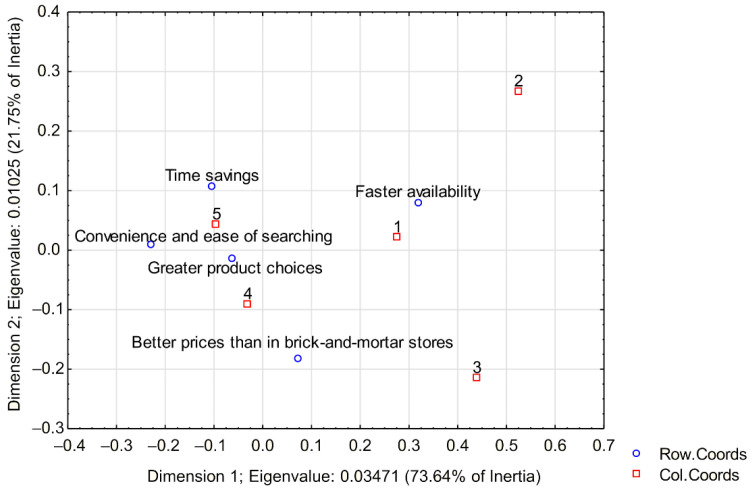
Correspondence map; variables What is your reason for online grocery shopping? and Level of agreement.

Furthermore, the reasons for buying organic food online were surveyed. The level of agreement was rated on a scale of 1 (disagree) to 5 (agree); see Figure 1. Most respondents buy organic food online because of the time savings, greater product choices, and convenience and ease of searching. A smaller proportion of respondents report better prices than in brick-and-mortar stores. Fewest respondents agree with faster availability when shopping for organic food online.

**Figure 2:**
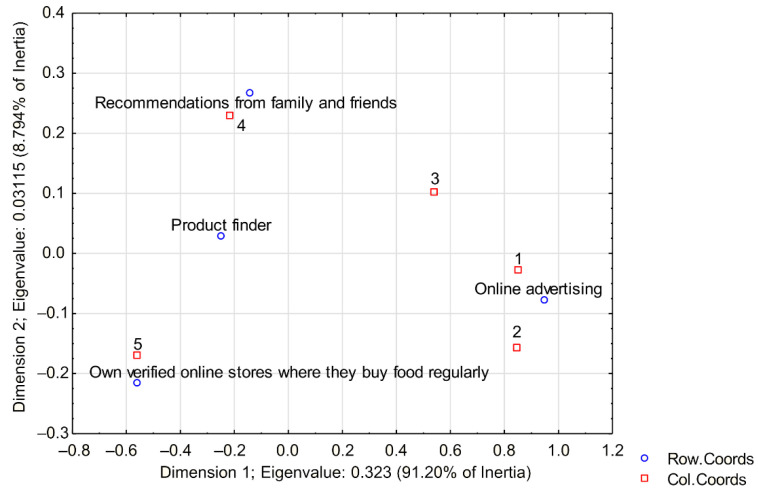
Correspondence map; variables How do you look for online grocery store? a Level of agreement.

An analysis of the use of communication media influencing organic food purchases was also conducted. The level of agreement was rated on a scale of 1 (disagree) to 5 (agree); see Figure 2. Most respondents use recommendations from family and friends. A large proportion of respondents also already have their own verified online stores where they buy organic food regularly.

## 4. Discussion

Our research shows that most respondents shop for groceries online in order to save time, have a wider choice of products and due to more convenient and easier searching. This statement is consistent with the views of many authors (e.g., [49,50,51]) and such progress can be expected in the future [73], because digitalization plays a crucial role [74]. Even some studies found that virtual reality technology is effective for online shopping [75]. According to [76], augmented reality and virtual reality technologies have been used to improve the online shopping experience. As we are concentrating on organic food, consumers trust organic food and organic certifications in European countries [52,77]. The European Union pushes for the development of ecological agriculture and supports it politically [78,79]. Even organic farming is energy efficient, and less or no fertilizers are used [80,81].

According to [33], more and more men go shopping as more women work out of their homes. However, our analysis shows that the proportion of respondents shopping for groceries online is not significantly related to gender. Roughly 80% of both men and women occasionally shop for groceries online. Conversely, the frequency of online grocery shopping increases with the higher education of the respondents. These results are not shared with research presented in [82], which did not find a relationship between the frequency of organic food purchases and education. According to the authors, gender, household income and family size also have no effect on the frequency of purchases. In contrast, ref. [83] concluded that factors that affect the frequency of organic food purchases include, but are not limited to, age, income, education, and the size of the community in which the respondents live.

The results of [36] suggest that there is a relationship between the age of customers and online grocery shopping. According to [37], older people tend to be more loyal due to the fact that they find it difficult to cope with changes in the store environment. However, our research shows that the dependence of the fact that the respondent shops for groceries online on age is not statistically significant, yet there are some differences. Almost 90% of the respondents in the 26–35 and 36–45 age categories shop for groceries online, while only less than 80% of younger or older respondents do so.

According to [38], household income has a significant impact on purchasing decisions. Our study confirms that the higher the respondents’ net monthly income, the more often they buy organic food online.

Research findings suggest that relative proximity to home and lower prices are the main factors influencing grocery shopping in brick-and-mortar stores [42]. This is not consistent with our research, as the proportion of respondents shopping for groceries online does not have a clear trend depending on the size of the municipality in which the respondent lives. Moreover, according to [84], distance of residence from the store does not affect whether people shop online.

The authors of [42] found that household size is related to unplanned purchases, which is positively related to the size of the grocery bill. If the household is large, consumption will also be high, which may indicate a relationship between organic food consumption and household loyalty. Current research confirms that family size has a positive effect on organic food purchases (e.g., [43]). Our results show a majority decreasing trend in the frequency of online organic food shopping as the number of household members increases. Consumers with more than four household members purchase organic food on the Internet least frequently. Single-person households are the most likely to buy organic food online. The same is confirmed in [84], as the conclusion is that smaller households buy organic food online more often.

A more comprehensive study in this area [45] found that shoppers’ age, gender, education, occupation, monthly household income, family size and distance travelled to the store were significantly related to retail choice decisions. Our research reveals that the proportion of respondents shopping for groceries online does not have a clear trend depending on the size of the municipality in which the respondent lives.

On the limitations of the study, to find out the consumer profile of a typical customer, identification variables were chosen—gender, age, education, household income, number of children in the household and number of household members. There are other key important factors that could be included in the next research, such as, e.g., product prices, market distance and possibly the cost of transport, the amount of time available, the sensitivity to the environment and the perception of health risk [85,86,87]. In addition, a limitation of this study is that the study data set is during the COVID-19 period (September 2020 to December 2020), and the study is only for the Czech Republic. A motivation for authors doing future research should be to include studying the situation after the COVID-19 pandemic and focusing on more socio-demographic characteristics of consumers.

## 5. Conclusions

Out of the surveyed sample of respondents, 80.58% of them shop for organic food on the Internet. Several identifying variables were selected and their effect on the frequency of online grocery shopping was examined using different statistical methods. These were discrete or categorical variables, and logistic regression was therefore used for the overall model and contingency table analysis for the partial dependencies. In the overall model, there was a significant effect of the variables of education, income and number of household members. According to the sign of the regression coefficients, the frequency of internet purchases increases significantly with the higher education and income of the respondents, while it decreases with increasing the number of household members. A more detailed description of these dependencies was obtained by contingency tables analysis. In all cases, significant statistical dependence was found using a Pearson’s chi-square test, and the intensity of dependence was measured by a Pearson’s contingency coefficient. This was mostly a moderate intensity of dependence and the coefficient was always statistically significant. Overall, the frequency of internet purchases was found to increase with the higher education of the respondents, from values roughly over 77% for those who finished high school to 88% for respondents with a university degree. According to the analysis of the residuals, university-educated respondents have the largest contribution to the existence of dependence. The frequency of online purchases further increases with the higher income of respondents, from around 72% for respondents with a monthly family income up to CZK 20,000 to 86% for those who earn above CZK 50,000 per month. Respondents with the highest and lowest incomes have the largest share in the emergence of this dependency. The frequency of internet purchases decreases with a larger household, from around 90% for respondents living alone to 68% for individuals from families of five or more, which have also been shown to have the greatest impact on the dependency. When the reasons for internet shopping were investigated, it was shown that most respondents shop for organic food online to save time, to have a wider choice of products and to make searching more convenient and easier. When asked about the method of finding an online store, respondents most frequently cited recommendations from family and friends and verified online stores. The aim of our paper was to create a profile of a typical online grocery shopper focusing on organic food. This paper and the research data could be used by marketing professionals, mainly the coordinators of the brands of origin and quality and mainly the coordinators of the BIO label in the Czech Republic, which is a part of the Czech ministry of Agriculture.

## Figures and Tables

**Table 1:** Relative distribution of respondents in individual categories.

Variables	Values	Percentage
Sex	Women	65.13%
	Men	34.87%
Net monthly family income	Up to 20,000 incl.	12.68%
	20,001–30,000	18.23%
	30,001–40,000	24.31%
	40,001–50,000	19.82%
	50,001 and more	24.97%

Source: (Authors’ calculations).

**Table 2:** Regression model parameters.

	Coefficient	Standard Deviation Error	Wald’s Statistics	Significance.	Lower 95%	Upper 95%
Constant	0.93	0.36	6.68	0.0098	0.23	1.65
Your highest level of education	0.28	0.13	4.73	0.0296	0.03	0.52
Net monthly income of your household	0.27	0.08	12.67	0.0004	0.12	0.41
How many members is in your household?	−0.25	0.08	8.97	0.0028	−0.41	−0.09

Source: (authors’ calculations).

**Table 3:** Columnar relative frequencies (variables online grocery shopping and highest education of respondents).

Column %	Apprenticeship, Elementary	High School	University
No	22.58%	19.08%	11.70%
Yes	77.42%	80.92%	88.30%

Source: (authors’ calculations).

**Table 4:** Adjusted standardized residuals, threshold 2.638 (variables online organic food shopping and highest education).

		Level of Education		
		Apprenticeship, Elementary	High School	University
online shopping	no	2.5	−0.1	−2.9
	yes	−2.5	0.1	2.9

Source: (authors’ calculations).

**Table 5:** Columnar relative frequencies (variables online organic food purchases and net monthly family income), in CZK.

Column%	Up to 20,000 incl.	20,001–30,000	30,001–40,000	40,001–50,000	50,001 and More
No	28.13%	19.57%	23.91%	15.33%	13.76%
Yes	71.88%	80.43%	76.09%	84.67%	86.24%

Source: (authors’ calculations).

**Table 6:** Adjusted standardized residuals, cut-off value 2.807 (variables online organic food purchases and monthly family income), in CZK.

		Net Monthly Family Income				
		Up to 20,000 incl.	20,001–30,000	30,001–40 000	40,001–50,000	50,001 and More
online shopping	no	2.3	0.0	1.8	−1.4	−2.3
	yes	−2.3	0.0	−1.8	1.4	2.3

Source: (authors’ calculations).

**Table 7:** Columnar relative frequencies (variables online organic food purchases and number of persons in the household).

Column %	1	2	3	4	5 and More
No	10.53%	19.21%	20.92%	16.74%	31.52%
Yes	89.47%	80.79%	79.08%	83.26%	68.48%

Source: (authors’ calculations).

**Table 8:** Adjusted standardized residuals, cut-off value 2.807 (variables online organic food purchases and number of persons in the household).

		Members				
		1	2	3	4	5 and More
online shopping	no	−2.1	−0.1	0.5	−1.2	3.1
	yes	2.1	0.1	−0.5	1.2	−3.1

Source: (authors’ calculations).

## Data Availability

Not applicable.
